# A Systematic Review of Fall Risk Factors in Stroke Survivors: Towards Improved Assessment Platforms and Protocols

**DOI:** 10.3389/fbioe.2022.910698

**Published:** 2022-08-08

**Authors:** Masoud Abdollahi, Natalie Whitton, Ramin Zand, Mary Dombovy, Mohamad Parnianpour, Kinda Khalaf, Ehsan Rashedi

**Affiliations:** ^1^ Industrial and Systems Engineering Department, Rochester Institute of Technology, Rochester, NY, United States; ^2^ Department of Neurology, Geisinger Neuroscience Institute, Danville, PA, United States; ^3^ Department of Rehabilitation and Neurology, Unity Hospital, Rochester, NY, United States; ^4^ Department of Mechanical Engineering, Sharif University of Technology, Tehran, Iran; ^5^ Department of Biomedical Engineering, Khalifa University of Science and Technology, and Health Engineering Innovation Center, Abu Dhabi, United Arab Emirates

**Keywords:** stroke, fall risk factors, fall risk assessment, cost-benefit analysis, dual-task paradigm, performance assesment, detailed motion analysis

## Abstract

**Background/Purpose:** To prevent falling, a common incident with debilitating health consequences among stroke survivors, it is important to identify significant fall risk factors (FRFs) towards developing and implementing predictive and preventive strategies and guidelines. This review provides a systematic approach for identifying the relevant FRFs and shedding light on future directions of research.

**Methods:** A systematic search was conducted in 5 popular research databases. Studies investigating the FRFs in the stroke community were evaluated to identify the commonality and trend of FRFs in the relevant literature.

**Results:** twenty-seven relevant articles were reviewed and analyzed spanning the years 1995–2020. The results confirmed that the most common FRFs were age (21/27, i.e., considered in 21 out of 27 studies), gender (21/27), motion-related measures (19/27), motor function/impairment (17/27), balance-related measures (16/27), and cognitive impairment (11/27). Among these factors, motion-related measures had the highest rate of significance (i.e., 84% or 16/19). Due to the high commonality of balance/motion-related measures, we further analyzed these factors. We identified a trend reflecting that subjective tools are increasingly being replaced by simple objective measures (e.g., 10-m walk), and most recently by quantitative measures based on detailed motion analysis.

**Conclusion:** There remains a gap for a standardized systematic approach for selecting relevant FRFs in stroke fall risk literature. This study provides an evidence-based methodology to identify the relevant risk factors, as well as their commonalities and trends. Three significant areas for future research on post stroke fall risk assessment have been identified: 1) further exploration the efficacy of quantitative detailed motion analysis; 2) implementation of inertial measurement units as a cost-effective and accessible tool in clinics and beyond; and 3) investigation of the capability of cognitive-motor dual-task paradigms and their association with FRFs.

## 1 Introduction

Stroke is currently considered as the second leading cause of mortality for individuals above the age of 60 years, and the fifth leading cause of death in individuals aged 15–59 years old, worldwide (Thrift et al., 2017). According to the World Health Organization (WHO), every year, 15 million people are diagnosed with stroke, of which, approximately 6 million die and another 5 million are left with permanent disabilities (CDC, 2019) (National library of medicine, 2022) (Thomas et al., 2000). In the United States, more than 795,000 people suffer a stroke every year, leading to an annual financial burden of nearly $46 billion, including the cost of health care services, medications, and missed days of work. Falling is a very common complication consequent to stroke, where both physical (weakness, paralysis, sensory disturbances, and impaired postural control) and mental impairments (mental fatigue, depression and impaired cognitive function) associated with stroke can contribute to regular falls (Larén et al., 2018). Moreover, most stroke patients, especially those who have suffered ischemic strokes, are prescribed antiplatelets or anticoagulants for secondary stroke prevention, which could increase their propensity for post-trauma and bleeding upon falling. Indeed, falls are seven times more prevalent among this population in comparison to healthy individuals and are often more consequential (Langhorne et al., 2000; Weerdesteyn et al., 2008; Melillo et al., 2015; Wei et al., 2017). Research studies have indicated that approximately half of stroke patients experience at least one fall in the first year post-stroke and that falling leads to further morbidity and mortality along with a dramatic increase in the cost of care (Andersson et al., 2006; Mackintosh et al., 2006; Ashburn et al., 2008; Kerse et al., 2008; Baetens et al., 2011; Simpson et al., 2011; Blennerhassett et al., 2012; Tilson et al., 2012; Yoshimoto et al., 2016). Moreover, falling often causes hip fractures and various other motion restrictions, which poses limitations on performing activities of daily living (ADLs) (Pouwels et al., 2009). This, in turn, triggers a viscous cycle in terms of the effect of immobility on the musculoskeletal system, leading to further compromise in musculoskeletal health and mobility of stroke patients and hence an increased risk of falls. It is, therefore, imperative to assess the risk of falling among stroke survivors during their path to partial or full recovery leverage the information towards more effective predictive and rehabilitation strategies.

In the last two decades, several studies have targeted fall risk assessment among stroke survivors (Ashburn et al., 2008; Baetens et al., 2011; Allin et al., 2016; An et al., 2017). More specifically, a growing body of literature has been exploring the association between the different fall risk factors (FRFs) and the frequency of falls. Risk factors are typically categorized into different groups, including demographics, medications, physical capability, cognitive impairments, among others. For example, the outcome of timed up and go (TUG) testing, during which the participant is asked to stand up from a chair, walk for three meters, return, and again sit on the same chair, can be considered as a FRF (Podsiadlo and Richardson, 1991; Jalayondeja et al., 2014; Pinto et al., 2016). Overall, most studies on fall risk assessment of stroke survivors follow a common approach, whereby several risk factors are identified to generate an initial pool of factors to be analyzed. Implementing statistical analysis tools, these factors are then compared between stroke patients who have fallen and those who have not. The published articles in this area indicate that there are more than 100 FRFs which can be included in the initial pool. However, due to the large number and variety of risk factors, it is not feasible to consider all of them in one study, which makes it challenging to select the appropriate initial list of FRFs.

To the best of our knowledge, only three review papers on fall risk assessment within the stroke community were published prior to this study. Walsh et al. (2016) conducted a systematic review on fall risk prediction models for stroke patients. Their results discussed several models to assess fall risk, recommending that future studies need to focus on the validation and improvement of current available models. In another study, Tan and Tan. (2016). used a narrative review to explore the epidemiology of falls within the stroke community. They categorized the risk factors for the elderly stroke population into several groups, including motor deficits, cognitive function, medication, and psychological risk factors, concluding with suggestions for fall prevention and management strategies for stroke survivors. Xu et al. (2018) conducted a meta-analysis of common FRFs to identify the most significant ones leading to falls. Their findings indicated that impaired mobility, reduced balance, use of sedative/psychotropic medications, disability in self-care, depression, cognitive impairment, and history of prior falls all had strong associations with falls among stroke survivors. One of the main limitations of this meta-analysis, on the other hand, was the insufficient number of observational studies meeting the requirement to be involved in the analysis of each risk factor. Their findings indicated that impaired mobility and reduced balance, with odds ratios (OR) of 4.36 and 3.87, respectively, proved to be the most impactful factors for falls among stroke survivors (Xu et al., 2018). It must be noted, however, that the reported OR data were calculated from only three studies, since the authors excluded a significant portion of studies due to the high heterogeneity, as well as lack of providing OR data in their results. The characteristics of this study, therefore, cast some uncertainty as to the conclusions regarding the most significant fall risk factors despite shedding light on this relevant issue. Hence, although numerous studies were conducted to identify FRFs for stroke survivors, further work incorporating less heterogeneity and proper outcome assessment (e.g., involving OR) is still needed to validate the association of common FRFs with fall occurrence for the stroke community.

To bridge the aforementioned knowledge gap in literature, researchers need to establish a standard reference point for identifying the appropriate FRFs to generate an initial pool of risk factors. To this end, this review will provide a commonality analysis on the FRFs and their significance ratio among the articles considering each factor. Furthermore, the articles will be explored to categorize their considered FRFs into subjective and objective classes. Finally, the articles will be reviewed to identify the trend of implementing motion analysis to identify the risk of fall in stroke community. Briefly, the results of this study will help: 1) to provide evidence-based data by which researchers can determine which FRFs to include in the initial pool of factors; 2) to explore changes over time regarding the most salient FRFs as described by researchers; and 3) to identify the potential opportunities such as conducting detailed motion analysis employing IMU sensors while performing cognitive-motor dual-task to improve the quality of fall risk assessment among stroke survivors. The outcome of this study may facilitate the development of a more efficient and accurate approach/model to conduct future fall risk assessment studies.

## 2 Methods

### 2.1 Search Strategy

In order to conduct the review, the guidelines for Preferred Reporting Items for Systematic review and Meta-analyses (PRISMA) were implemented (Page et al., 2021). A systematic review approach was developed to identify the relevant articles for the review. We identified the potentially eligible studies by systematically searching the databases: MEDLINE, EMBASE, Web of Science, and PubMed. The search queries were primarily conducted from 1995 until 2020, without restriction on study design, document type, or language. Specifically, the title, abstract, and keywords in potentially relevant articles were searched for specific words: (“stroke” OR “cerebrovascular accident” OR “cerebrovascular apoplexy” OR “cerebrovascular disease” OR “cerebrovascular stroke”) AND (“falls” OR “falling”) AND (“prospective” OR “follow up” OR “cohort” OR “case-control” OR “longitudinal study” OR “cohort study” OR “observational study” OR “case-control study”). The entry terms for each keyword were extracted implementing the Medical Subject Heading (MeSH) tool. The same search terms and Boolean combinations were used to identify the relevant articles while using the advance search in each database. We augmented the search results by manually forward and backward (in Google Scholar) citation tracking. Additional articles were added from the authors’ archives or through cross-referencing.

### 2.2 Study Selection

Studies addressing the prediction of fall risk and/or those identifying significant risk factors among stroke survivors were recorded. Since the focus of this review was to evaluate the factors involved in the FRFs among stroke patients, any prospective study describing a follow-up period of more than one day was included. Two authors independently searched the literature and merged their results, and then the final selected studies were carefully reviewed to ensure that all the articles met the inclusion criteria for the review. The inclusion criteria consisted of: 1) a prospective study on stroke survivors; 2) assessing the risk of fall; 3) published in English. Studies were excluded if participants had other neurological diseases, such as Alzheimer, Parkinson’s disease, multiple sclerosis, and spinal cord injury.

### 2.3 Data Extraction

The results of the comprehensive database search were screened for relevance, and the selected papers were analyzed. The data from each study were extracted using a format developed by the authors. The form included the following information from each article: Authors, Publication date (year), Location of the study, Sample size, Setting, Number of Parameters, Labeling of the Participants, Significant Factors, Non-Significant Factors, Univariate/Bivariate analysis: Output Type, Multivariate Analysis (Model): Method, Age of the participants, and Notes.

### 2.4 Risk of Bias

Most fall risk assessment studies could be categorized as cohort studies which fall under the umbrella of observational studies. Considering the various available tools to assess the risk of bias in observational studies (Deeks et al., 2003), the Cochrane Tool to Assess Risk of Bias in Cohort Studies was selected (Higgins, 2008). The tool includes eight questions with 4 choices each. One of the questions was not applicable in fall risk assessment studies and consequently removed from the analysis. The remaining questions were related to the selection of the cohorts, comparability, and assessment of outcome. The seven questions were answered for each of the 27 articles by two authors (i.e., MA and ER) independently. The cases of discrepancy in the results in terms of risk of bias assessment for each paper were addressed in a meeting between the two authors. Since there were seven questions with four possible answers reflecting the quality of the study in various aspects (ranging from 0 for high risk of bias to 3 for low risk of bias), the overall score for each study was between 0 and 21. Studies with scores of <14, 14 to 17, and >17 were classified as low, acceptable, and good quality, respectively.

### 2.5 Fall Risk Factor Commonality and Significance Analysis

As noted earlier, it was not feasible to conduct a thorough meta-analysis on the various FRFs due to the limited number of studies for each FRF required for such an analysis. This impediment stems from discrepancies in the heterogeneity of factors, as well as discrepancies in the format of outcomes—principally involving a variety of preferred statistical measures. To address these issues, we have developed a list of significant risk factors towards affording researchers better insight into the commonality and significance of the factors. Using this risk factor-specific approach, we have reported the number of studies considering each factor, as well as the number of studies in which that particular factor was determined to be significant. In order to simplify the process of comparing factors, a scale was created for each factor to calculate the ratio of the number of studies which identified a given FRF to be a significant to the number of the studies considering that factor.

Significance Ratio(SR)=Number of the studies finding the FRF to be significant in fall risk Number of the studies considering the FRF

Note that if at least one of the items in a particular category had a significant impact on the fall risk, the category was classified as a significant factor. As an example, Mansfield et al. conducted a balance test requiring participants to stand as still as possible on a force plate. They subsequently calculated the mediolateral root mean square (RMS) of the center of pressure (COP), identifying it as a significant risk factor for falls, in contrast, to the anteroposterior RMS of COP which was determined to be non-significant. Therefore, since there was a factor from the category of balance-related measures among the significant FRFs, we considered that category (i.e., balance-related measures) as significant in that study.

### 2.6 Analysis of the Balance and Mobility-Related Factors in Fall Risk

This study also analyzed changes over time in the methods assessing the balance and mobility of stroke patients and how that information factored into the fall risk assessment process. Accordingly, any articles considering these factors were further analyzed to determine all implemented approaches. Initially, the identified balance and mobility-related factors from literature were categorized as either subjective or objective. We have also classified objective balance and mobility-related factors according to the equipment used for assessment, which provides researchers with a more accurate depiction of the level of motion analysis achieved in the various studies. For example, TUG testing typically measures the time for accomplishing a specific physical task, notably standing from/sitting on a chair, walking, and turning. Since the only equipment measuring the output of this test (i.e., time) is a timer, we can conclude that it does not pay sufficient attention to more detailed motion analysis parameters such as gait characteristics. However, a similarly designed study might utilize a force plate or inertial measurement unit (IMU) sensors which can deliver more detailed gait-analysis data, potentially improving the accuracy of identifying the risk for falls among stroke victims.

## 3 Results

### 3.1 Study Identification

**FIGURE 1:**
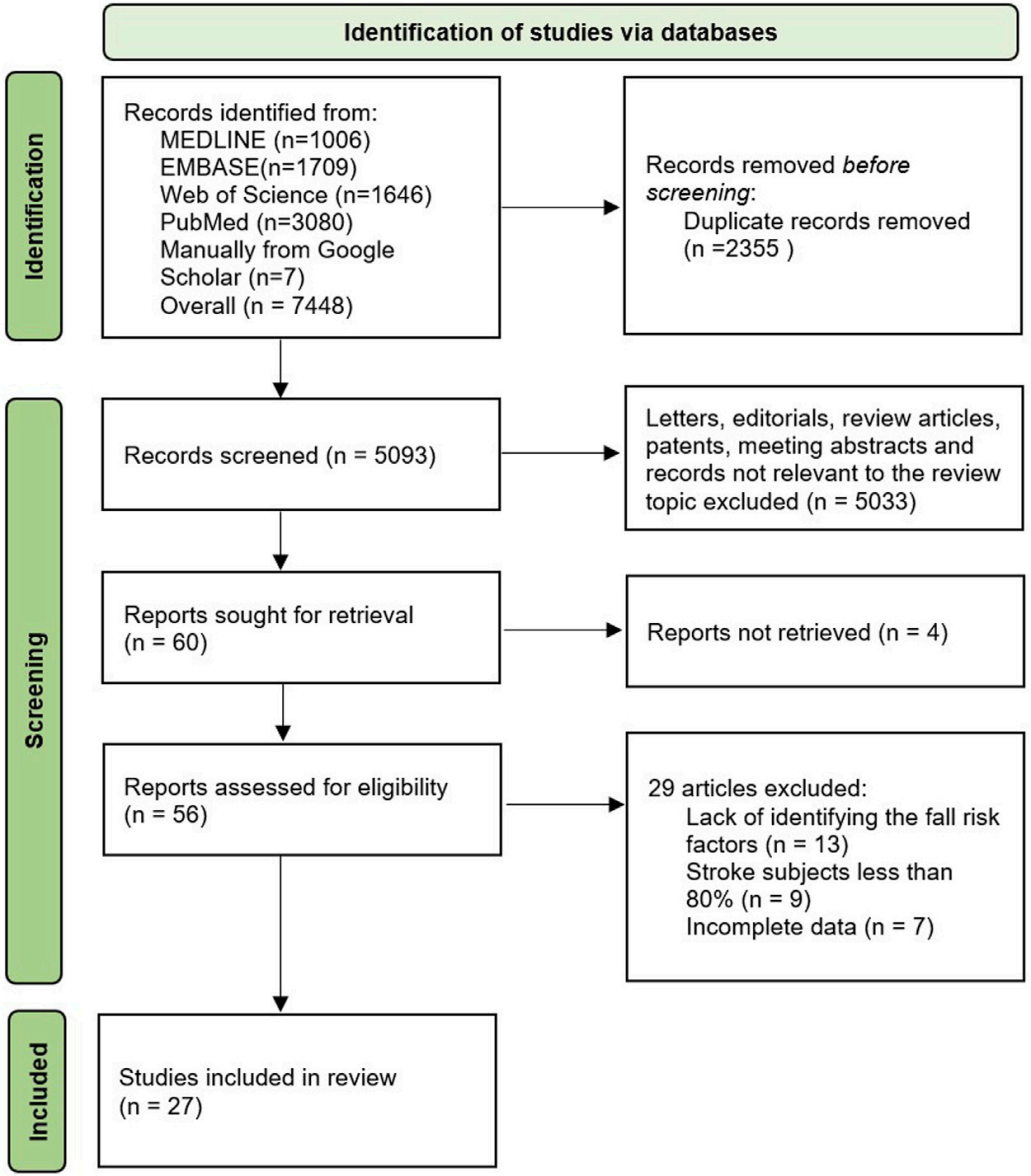
Identification of the eligible studies to be included in the review.

A total of 10,746 articles were initially identified through the process of searching the databases and relevant repositories as described above. Due to duplication and not meeting the inclusion criteria, a portion of these articles were removed, resulting in a total of 27 articles considered relevant (Figure 1).

### 3.2 Study Characteristics

The characteristics of interest included publication year, location, sample size, setting, and details of the patient labeling, as shown in Table 1.

**TABLE 1:** The characteristics of the reviewed articles.

Article	Location	Sample size	Setting	Follow-up details
Nyberg and Gustafson, (1997)	Sweden	135	Hospital stay/2–4 weeks after the stroke/after acute phase/stroke rehabilitation unit	8 weeks
Yates et al. (2002)	United States	280	Data collected in 3–14 days of stroke onset.	1, 3, and 6 months
Lamb et al. (2003)	United States	94	At home	12 months
Olsson et al. (2005)	Sweden	158	Hospital stay/2–4 weeks after the stroke/after acute phase/stroke rehabilitation unit	8 weeks
Mackintosh et al. (2006)	Australia	55	Community	6 months
Wada et al. (2007)	Japan	101	Community	12 months
Ashburn et al. (2008)	United Kingdom	115	Community	12 months
Kerse et al. (2008)	New Zealand	1,104	Community	6 months
Divani et al. (2009)	United States	1,174	Community	24 months
Maeda et al. (2009)	Japan	72	Admitted to rehabilitation center	Variant
Persson et al. (2011)	Norway	96	Rehabilitation hospital and community	12 months
Simpson et al. (2011)	Canada	80	Community	13 months
Nyström and Hellström, (2013)	Sweden	68	Acute stroke unit/newly diagnosed with stroke	6 weeks
Alemdaroğlu et al. (2012)	Turkey	66	Rehabilitation hospital, then home	6 months
Blennerhassett et al. (2012)	Australia	30	Community	6–36 months
Jalayondeja et al. (2014)	Thailand	97	Stroke patients enrolled within 1 month of their stroke/outpatients/fall at home or outside	6 months
Breisinger et al. (2014)	United States	419	Admitted to rehabilitation unit	Variant
Callaly et al. (2015)	Ireland	522	Community	24 months
Mansfield et al. (2015)	Canada	95	Rehabilitation hospital and community	6 months
Goljar et al. (2016)	Slovenia	232	Admitted for the first time to the stroke rehabilitation ward/stroke patients during their first inpatient rehabilitation	12 months
Pinto et al. (2016)	Brazil	131	Outpatients in stroke clinic	2 years
Taylor-Piliae et al. (2016)	United States	10	Mix of outpatients and post-stroke at least 3 months after stroke	2 days
Yoshimoto et al. (2016)	Japan	65	Patients discharged from a rehabilitation ward	12 months
Wei et al. (2017)	Taiwan	112	Hospital or rehabilitation ward and then community	6 months
Lee and Jung, (2017)	South Korea	71	Community	12 months
Foster et al. (2018)	United Kingdom	7,267	Immediate data from hospitalized patients	10 years
Persson et al. (2018)	Sweden	504	Stroke unit at hospital	4 days

Assessment of the risk of bias indicated that all the articles in the review had at least acceptable quality based on formula described earlier. The detailed results of this analysis are shown in Table 2.

**TABLE 2:** Risk of bias assessment using Cochrane Tool to Assess Risk of Bias in Cohort Studies.

Article	1 Was selection of exposed and non-exposed cohorts drawn from the same population?	2 Can we be confident in the assessment of exposure?	3 Did the study match exposed and unexposed for all variables that are associated with the outcome of interest or did the statistical analysis adjust for these prognostic variables?	4 Can we be confident in the assessment of the presence or absence of prognostic factors?	5 Can we be confident in the assessment of outcome?	6 Was the follow up of cohorts adequate?	7 Were co-interventions similar between groups?	Sum	Quality
Nyberg and Gustafson, (1997)	3	2	3	2	3	2	2	17	Good
Yates et al. (2002)	3	2	2	2	3	3	2	17	Good
Lamb et al. (2003)	3	2	3	2	1	3	2	16	Acceptable
Olsson et al. (2005)	3	2	3	2	3	2	2	17	Good
Mackintosh et al. (2006)	3	2	3	2	2	3	2	17	Good
Wada et al. (2007)	3	2	3	2	3	3	2	18	Good
Ashburn et al. (2008)	3	2	3	2	3	3	2	18	Good
Kerse et al. (2008)	3	2	3	2	3	3	2	18	Good
Divani et al. (2009)	3	2	3	2	3	3	2	18	Good
Maeda et al. (2009)	3	2	2	2	2	2	2	15	Acceptable
Persson et al. (2011)	3	2	3	2	3	3	2	18	Good
Simpson et al. (2011)	3	2	3	2	3	3	2	18	Good
Anna and Karin (2012)	3	2	2	2	2	2	2	15	Acceptable
Alemdaroglu et al. (2012)	3	2	3	2	3	3	2	18	Good
Blennerhassett et al. (2012)	3	2	2	2	3	3	2	17	Good
Jalayondeja et al. (2014)	3	2	3	2	2	3	2	17	Good
Breisinger et al. (2014)	3	2	3	3	3	2	2	18	Good
Callaly et al. (2015)	3	2	3	2	2	3	2	17	Good
Mansfield et al. (2015)	3	2	3	3	2	3	2	18	Good
Goljar et al. (2016)	3	2	3	2	1	3	2	16	Acceptable
Pinto et al. (2016)	3	2	2	2	3	3	2	17	Good
Taylor-Pilliae et al. (2016)	1	3	2	3	2	1	2	14	Acceptable
Yoshimoto et al. (2016)	3	2	2	2	3	3	2	17	Good
Wei et al. (2017)	3	2	3	3	2	3	2	18	Good
Lee and Jung, (2017)	3	2	3	3	2	3	2	18	Good
Foster et al. (2018)	3	2	3	3	2	2	2	17	Good
Persson et al. (2018)	3	3	3	2	2	2	2	17	Good

The frequency for the 14 most common FRFs assessed in the articles is shown in Table 3. The FRFs were classified into six categories: sociodemographic risk factors, sensorimotor risk factors, cognitive risk factors, psychosocial risk factors, medical risk factors, and balance and mobility risk factors.

**TABLE 3:** Commonalities among the 14 most significant FRFs in the articles.

Risk factors	Number of studies considering the factor	Number of studies in which the factor was significant	Significance ratio (%)	References
Age	21	8	38	Nyberg and Gustafson, (1997); Yates et al. (2002); Mackintosh et al. (2006); Wada et al. (2007); Ashburn et al. (2008); Kerse et al. (2008); Divani et al. (2009); Maeda et al. (2009); Persson et al. (2011); Simpson et al. (2011); Alemdaroğlu et al. (2012); Breisinger et al. (2014); Jalayondeja et al. (2014); Callaly et al. (2015); Goljar et al. (2016); Pinto et al. (2016); Yoshimoto et al. (2016); Lee and Jung, (2017); Wei et al. (2017); Foster et al. (2018); Persson et al. (2018)
Gender (female)	21	5	24	Nyberg and Gustafson, (1997); Yates et al. (2002); Olsson et al. (2005); Mackintosh et al. (2006); Wada et al. (2007); Ashburn et al. (2008); Kerse et al. (2008); Divani et al. (2009); Maeda et al. (2009); Persson et al. (2011); Simpson et al. (2011); Breisinger et al. (2014); Jalayondeja et al. (2014); Callaly et al. (2015); Goljar et al. (2016); Pinto et al. (2016); Yoshimoto et al. (2016); Lee and Jung, (2017); Wei et al. (2017); Foster et al. (2018); Persson et al. (2018)
History of fall	9	4	44	Nyberg and Gustafson, (1997); Mackintosh et al. (2006); Ashburn et al. (2008); Kerse et al. (2008); Divani et al. (2009); Alemdaroğlu et al. (2012); Callaly et al. (2015); Pinto et al. (2016); Foster et al. (2018)
Motor function/impairment (lower Extremities)	17	11	65	Nyberg and Gustafson, (1997); Yates et al. (2002); Lamb et al. (2003); Olsson et al. (2005); Wada et al. (2007); Ashburn et al. (2008); Divani et al. (2009); Maeda et al. (2009); Persson et al. (2011); Alemdaroğlu et al. (2012); Nyström and Hellström, (2013); Jalayondeja et al. (2014); Callaly et al. (2015); Goljar et al. (2016); Pinto et al. (2016); Lee and Jung, (2017); Wei et al. (2017)
Cognitive impairment	11	4	36	Nyberg and Gustafson, (1997); Lamb et al. (2003); Wada et al. (2007); Kerse et al. (2008); Maeda et al. (2009); Simpson et al. (2011); Alemdaroğlu et al. (2012); Jalayondeja et al. (2014); Goljar et al. (2016); Wei et al. (2017); Persson et al. (2018)
Depression	8	4	50	Nyberg and Gustafson, (1997); Lamb et al. (2003); Olsson et al. (2005); Mackintosh et al. (2006); Kerse et al. (2008); Alemdaroğlu et al. (2012); Callaly et al. (2015); Wei et al. (2017)
fall Efficacy Scale (FES)	4	3	75	Mackintosh et al. (2006); Blennerhassett et al. (2012); Wei et al. (2017); Persson et al. (2018)
Visual impairment	7	3	43	Nyberg and Gustafson, (1997); Yates et al. (2002); Lamb et al. (2003); Olsson et al. (2005); Mackintosh et al. (2006); Divani et al. (2009); Alemdaroğlu et al. (2012)
Duration of stroke	8	3	38	Lamb et al. (2003); Wada et al. (2007); Ashburn et al. (2008); Divani et al. (2009); Maeda et al. (2009); Alemdaroğlu et al. (2012); Goljar et al. (2016); Pinto et al. (2016)
Stroke type	8	0	0	Yates et al. (2002); Wada et al. (2007); Kerse et al. (2008); Maeda et al. (2009); Breisinger et al. (2014); Jalayondeja et al. (2014); Goljar et al. (2016); Wei et al. (2017)
Urinary incontinence/medications	8	2	25	Nyberg and Gustafson, (1997); Lamb et al. (2003); Olsson et al. (2005); Divani et al. (2009); Persson et al. (2011); Alemdaroğlu et al. (2012); Callaly et al. (2015); Pinto et al. (2016)
Use of sedative/psychotropic medications	4	1	25	Nyberg and Gustafson, (1997); Olsson et al. (2005); Wada et al. (2007); Alemdaroğlu et al. (2012)
Balance-related measures	16	13	81	Nyberg and Gustafson, (1997); Lamb et al. (2003); Olsson et al. (2005); Mackintosh et al. (2006); Ashburn et al. (2008); Maeda et al. (2009); Persson et al. (2011); Simpson et al. (2011); Alemdaroğlu et al. (2012); Blennerhassett et al. (2012); Jalayondeja et al. (2014); Mansfield et al. (2015); Yoshimoto et al. (2016); Lee and Jung, (2017); Wei et al. (2017); Persson et al. (2018)
Motion-related measures	19	16	84	Nyberg and Gustafson, (1997); Lamb et al. (2003); Olsson et al. (2005); Mackintosh et al. (2006); Ashburn et al. (2008); Kerse et al. (2008); Maeda et al. (2009); Persson et al. (2011); Simpson et al. (2011); Alemdaroğlu et al. (2012); Blennerhassett et al. (2012); Jalayondeja et al. (2014); Mansfield et al. (2015); Pinto et al. (2016); Taylor-Piliae et al. (2016); Yoshimoto et al. (2016); Lee and Jung, (2017); Wei et al. (2017); Persson et al. (2018)

Table 4 provides the extracted data from articles that considered stability and motion-based factors, which were then categorized as either subjective or objective. Moreover, the objective factors were subsequently classified into two groups: 1) studies without force/motion sensors; 2) studies with force/motion sensors (i.e., involving detailed motion analysis).

**TABLE 4:** The stability and mobility-related risk factors in the articles.

Article	Stability & mobility		
	Subjective	Objective	
		Without force/motion sensors	With force/motion sensors (detailed motion analysis)
Nyberg and Gustafson, (1997)	Katz ADL (activities of daily living), Fugl-meyer		
Lamb et al. (2003)	Balance problems and ADL difficulties while performing various tasks such as walking, dressing, and toileting		
Olsson et al. (2005)	Katz ADL, Fugl-meyer		
Mackintosh et al. (2006)	BBS score	Fast gait speed and step test score	
Ashburn et al. (2008)	BBS score, nottingham extended ADL, Rivermead upper limb, rivermead total score, rivermead leg and trunk,rivermead gross function	Mean functional reach	
Kerse et al. (2008)	Barthel index, FAI score (activity)		
Maeda et al. (2009)	BBS score		
Persson et al. (2011)	BBS score, SwePASS	10 MWT, TUG	
Simpson et al. (2011)	BBS score, ABC: Activity-Specific Balance Confidence Scale	TUG, 6 MWT	
Alemdaroğlu et al. (2012)	Fugl-Meyer		
Blennerhassett et al. (2012)	Environmental analysis of mobility questionnaire (EAMQ)	6 MWT, Four Square Step Test (FSST), Step Test (ST)	
Jalayondeja et al. (2014)	BBS score, Barthel Index	Timed up & Go (s), 10-m walk test (m/s): preferred speed & maximum speed, 2-min walk test	
Mansfield et al. (2015)			Detailed analysis of COP and gait
Pinto et al. (2016)	Quality of life (EQ-5D)	Timed up and Go quartile	
Taylor-Piliae et al. (2016)		postural transition (PT) duration (in seconds), Gait speed (meters per second)	Aborted PT attempts (number per day), Steps (number), Duration of gait (% of total activity)
Yoshimoto et al. (2016)	Barthel Index	10-m walking speed (m/s), One-leg standing time of the affected side (s), One-leg standing time of the unaffected side (s)	
Wei et al. (2017)			Gait and balanced detailed parameters
Lee and Jung, (2017)	Korean modified barthel index, fugl-meyer assessment, BBS, functional ambulation category		Postural sway velocity: eye open/closed firm/soft surface
Persson et al. (2018)	SwePASS, Self-perceived impaired postural control (section 13 BBS), Self-perceived previous physical activity level was assessed using the Saltin-Grimby Physical Activity Scale		

## 4 Discussion

To the best of our knowledge, up to date, there is no comprehensive literature review on fall risk assessment among stroke survivors. The most recent review presented a relevant meta-analysis on the various FRFs impacting stroke population (Xu et al., 2018), but only included a handful of studies which met the data-analysis requirements. There is a need, therefore, for identifying FRFs and understanding associated commonalities and trends in recent fall risk assessment articles to develop effective interventions. Towards this we reviewed a total of 27 articles spanning almost 3 decades from multiple countries to identify the most frequently cited FRFs and the number of studies categorizing them as significant risk factors. The second objective of this review was to explore changes in how different researchers consider these FRFs and propose specific ones which could potentially provide better outcomes. Such analysis could determine how shifting to a new level of FRF-based research, in conjunction with detailed motion analysis, could assist in developing more accurate fall risk prediction and assessment models.

### 4.1 Common FRFs and Significance Ratios

According to the commonality table (Table 3), the most common FRFs, as described in literature, included age, gender, motion-related measures, balance-related measures, motor function/impairment, and cognitive impairment. The significance ratio for age, cognitive impairment, and gender were 38%, 36%, and 24%, respectively, which demonstrates the lack of consensus among studies regarding the impact of these factors on fall risk assessment. By contrast, the significance ratio for motion-related measures, balance-related measures, and motor function/impairment were 84%, 81%, and 65%, respectively. These percentages imply a consensus that these factors have a significant impact on fall risk among stroke survivors. The large significance ratio for motion-based measures is compatible with earlier findings (Xu et al., 2018).

Among the less common FRFs, such as history of falls, depression, visual impairment, and fall efficacy scale (FES), FES presented the highest significance ratio. Representing the level of fear of falling among stroke survivor, FES had a significance ratio of 75%, which is the third largest after motion- and balance-related measures with significance ratios of 84% and 81%, respectively. It is noteworthy that visual impairment with a 43% significance ratio was among the important FRFs, which also confirms the importance of factors associated with balance control. In fact, vison, along with proprioception and balance-control mechanisms in the inner ear, are the primary systems for maintaining human balance. Consequently, since visual impairment could affect balance, a stroke victim’s vision represents a potentially important factor in fall risk assessment. Moreover, an inclusive assessment of balance measures should represent the combined effects of vision, as well as other sensory systems and proprioception in fall risk. Hence, it is highly recommended that both balance and FES should be considered in the initial pool of FRFs in future studies. Finally, the FRFs with the least significance ratios were urinary incontinence/medications, use of sedative/psychotropic medications, duration of stroke, and stroke type. It is worthy to note, that among the eight articles which considered stroke type, none identified it as a significant fall risk factor (Table 1).

As mentioned, the most common significant factors among the pool of reviewed articles were balance and motion-related measures, thus confirming the critical importance of these measures in determining the risk for falls among stroke victims. The list of measures in the various reviewed articles revealed that numerous subjective and objective scales were used to evaluate balance and motion in stroke survivors (Table 4). For example, subjective tools such as Katz ADL, Fugl-Meyer, and the Barthel index were routinely implemented in the assessed studies (Nyberg and Gustafson, 1997; Olsson et al., 2005; Jalayondeja et al., 2014). In general, these approaches help to determine self-reported physical status of patients by asking them to report about the quality of their daily physical activities, or by subjectively evaluating their physical capabilities by a clinician using observational gait and balance assessment measures. By contrast, more objective measures such as 10 MWT, 6 MWT, TUG, and FSST must be administered by someone who is skilled in their use (Persson et al., 2011; Simpson et al., 2011; Blennerhassett et al., 2012). These tests usually measure the time needed to accomplish a physical task, such as walking for 10 meters (10 MWT).

It is expected that reviewing the utilization of different factors reported in earlier studies could lead to beneficial information in the fall risk assessment and hence inspire more effective management strategies of stroke survivors. For example, exploring the evolution of balance and motion-based tools confirmed that earlier studies focused more on the implementation of subjective versus objective tools. Specifically, articles dating from 1997 to 2014 (12 out 19 articles) used subjective tools to assess the balance and motion of stroke survivors (Table 4). Moreover, in some of these studies, subjective measures were combined with standard objective tests such as TUG and 10 MWT, which are designed to measure a single parameter, such as speed or time while the subject performs a specific task. As described earlier, TUG testing requires the patient to stand up from a normal chair, walk for 3 m, return, and sit on the chair again; the output of this test is the time required to complete the task. Clearly, this type of assessment does not provide detailed motion parameters, such as those obtained from instrumented force plates or motion sensors. In 2015, Mansfield et al. was the first research team to conduct a study that considered a detailed analysis of motion during quiet standing (i.e., balance testing) and walking (i.e., gait testing) (Mansfield et al., 2015). Later, Taylor-Pilliae et al. investigated the capability of a single motion sensor in motion-monitoring of stroke survivors over the course of 48 h during usual daily activities (Taylor-Piliae et al., 2016). It should be noted that this study was more of a feasibility analysis designed to assess the capacity of the system in identifying fall risk indicators during posture transition and gait; thus, subjective measures were not considered. To the best of our knowledge, the study by Lee and Jung. (2017) was the only one to integrate both subjective tools along with the balance related-measures from detailed motion analysis The results of this study revealed that postural sway velocity with eyes-closed on a soft surface outperformed other subjective and objective measures such as BBS, Fugle-Meyer Assessment, and weight-bearing asymmetry in fall prediction for post-stroke individuals. Finally, Wei et al. (2017) conducted a study to explore the correlation between gait and balance parameters with respect to falls among stroke survivors.

Overall, among the reviewed articles, there were only four studies involving detailed motion analysis designed to identify the effect of motion in fall risk assessment (Mansfield et al., 2015; Taylor-Piliae et al., 2016; Lee and Jung, 2017; Wei et al., 2017). These studies were among the seven most-recent reviewed articles, which implies a trend towards clarifying the role of motion in fall risk identification within the stroke community. These four studies are further explored in Section 4.2 to investigate the potential for improving the accuracy of fall risk assessment models *via* the implementation of a thorough motion analysis protocol of stroke survivors.

### 4.2 Opportunities to Improve Fall Risk Assessment

Considering the gaps in the existing literature may shed some light on the future directions for this important area of study. The initial pool of FRFs in future studies could be identified according to Table 3, coupled with other relevant reviews on the FRFs impacting the stroke community. According to the results of this review and (Xu et al., 2018), balance and motion-related measures represent the most common and significant fall risk factors. These measures can be categorized into three classes: 1) subjective, 2) objective without force/motion sensors, and 3) objective with force/motion sensors (detailed motion analysis). Most of the reviewed studies implemented subjective tools for assessing the balance and motion of participants. However, as explained earlier, researchers in this area, have recently started to pay more attention to objective assessment of balance and motion analysis, especially with the rapidly emerging smart wearable tools and technologies, which promise a paradigm shift in gait and balance quantification of various movement pathologies including stroke (Mohan et al., 2021). With respect to objective categories, it remains to be determined which class (i.e., with or without force/motion sensors) could lead to more accurate fall risk assessment among stroke survivors. Hence, conducting fall risk assessment studies which includes measures from all three classes would be of great assistance in guiding researchers and clinicians in determining the most appropriate platform for assessing fall risk.

To augment the numerous available tests for assessing balance and motion in stroke survivors, the effect of the data type (i.e., subjective or objective) of a given system for capturing balance/motion data needs to be explored. Table 5 lists studies which incorporated a detailed balance and motion analysis and the systems/sensors which they utilized. The results indicate that IMUs, force plates, and pressure mats were typically implemented to investigate balance and gait. Force plates were mostly intended to calculate the location of the center of pressure (COP), as well as to determine balance-related parameters, such as the range of trajectory of the COP in different directions during quiet standing. Due to the fact that force plates typically limit data collection to a laboratory environment, utilizing wearable IMU sensors for motion and balance analysis is preferred since these sensors can be implemented outside a controlled environment; indeed, balance assessment using IMU sensors is a well-established approach (Mancini et al., 2011). The same techniques could be implemented to evaluate balance in stroke survivors using IMU sensors, where many potential locations can be identified for IMU sensor placement while collecting data from a participant in clinical settings and/or during ADLs. Accordingly, a recommended future study would involve identifying the optimal location(s) for IMU senor(s) placement with which the highest accuracy for fall risk assessment could be achieved.

**TABLE 5:** The articles conducted detailed balance and motion analysis and their implemented sensors.

The study	The analysis	Implemented sensors for data collection
Mansfield et al. (2015)	Detailed analysis of center of pressure and gait	Force plate & Pressure mat
Taylor-Piliae et al. (2016)	Postural transition (PT) duration (in seconds), gait speed (meters per second), aborted PT attempts (number per day), steps (number), duration of gait (% of total activity)	IMU sensor on chest
Wei et al. (2017)	Detailed analysis of center of pressure and gait	IMU sensor (location not clarified) & load sensors in the shoes
Lee and Jung, (2017)	Detailed analysis of center of pressure	Force plate

Another area within the scope of improvement of fall risk assessment studies involves the type of tasks during which motion data is collected. Importantly, although the significance ratio for cognitive impairment as an FRF was found to be 36%, cognitive factors may have larger impact on fall risk assessment when physical task performance is also factored in. For instance, dual-task paradigms are often utilized in fall risk assessment of the elderly population and patients suffering from multiple sclerosis (Wajda et al., 2013; Muir-Hunter and Wittwer, 2016; Rydalch et al., 2019; Rizzo et al., 2021). Likewise, there are many studies on the effect of cognitive-motor interference on the performance of stroke survivors (Haggard et al., 2000; Bowen et al., 2001; Cockburn et al., 2003; Hyndman et al., 2006; Kemper et al., 2006; Plummer-D’Amato et al., 2008; Dennis et al., 2009; Plummer-D'Amato et al., 2010; Plummer et al., 2013; Plummer et al., 2014). Specifically, it has been proven that when stroke survivors perform a cognitive task while walking, their gait speed (Bowen et al., 2001; Hyndman et al., 2006; Plummer-D’Amato et al., 2008), stride length (Hyndman et al., 2006; Plummer-D’Amato et al., 2008), cadence (Kemper et al., 2006; Plummer-D’Amato et al., 2008), and stride duration (Haggard et al., 2000; Cockburn et al., 2003; Plummer-D’Amato et al., 2008) all decrease. These findings are significant since most individuals are under some level of cognitive load while they are performing daily life activities. For example, remembering directions, listening to music, or chatting with a friend while walking are common across all communities and age brackets. Thus, in order to determine a more accurate fall risk assessment platform among stroke survivors, it would be best to assess performance using a dual-task paradigm. Cognitive-motor dual-task implementation would provide more realistic information about the functionality of stroke survivors during daily activities. Furthermore, it could transfer the performance quality of stroke survivors to some extreme region of difficulty, whereby discriminating between high fall risk (vs. low fall risk) individuals could be performed more easily. Such an approach could facilitate the development of fall risk assessment models with higher accuracy, especially in comparison to available models in the literature which rely on single-task paradigms for the functional assessment of stroke victims. Hence, future studies should prioritize dual-task paradigms for assessing the risk for falls within the stroke community.

Several methods could be implemented to execute cognitive-motor dual-task paradigm. To this end, there will be a physical task such as walking, TUG test, and balance test tied with a second task which is mainly to put a cognitive load on the stroke survivors. The physical task could be selected according to the objective of the studies. However, to put the cognitive load on the individuals, there are limited number of methods to be utilized. Generally, there are five categories of activities to be considered in dual-task paradigms. The first approach is the n-back tasks in which the subject is presented with a sequence of stimuli, and the task consists of indicating when the current stimulus matches the one from n steps earlier in the sequence (Voelcker-Rehage et al., 2006; Plummer-D’Amato et al., 2008; Coulacoglou and Saklofske, 2017). The load factor n can be adjusted to make the task more or less difficult. This method is basically putting the cognitive load through using working memory. Second method is the auditory clock which is associated with visuospatial cognition (Plummer-D’Amato et al., 2008; Kao and Pierro, 2021; Plummer et al., 2021). In this method, the participant hears a time (e.g., ‘‘two-oh-seven’’) and are asked to say ‘‘yes’’ if both hands are in a particular half of the clock and ‘‘no’’ if they are not. The spontaneous speech, auditory Stroop task, and counting backward are the other classes of activities to perform cognitive-motor dual-task (Brown and Marsden, 1991; Plummer-D’Amato et al., 2008). Among these, the researchers could select one or more of them and conduct the fall risk assessment study in stroke community. Since, it has not been investigated in the field of fall risk assessment in stroke survivors, comparison of the fall risk accuracy while using the mentioned methods and finding the most adequate method of implementing cognitive-motor dual-task paradigm could also be of a great help in the future studies.

There are several limitations in this review and the relevant literature which need to be improved in the future studies. The main drawback in the literature is that the studies did not provide the effect size for their analyses on the FRFs. Due to this gap in the literature, Xu et al., could not perform any analyses on the FRF with more than four articles which is a very few sample size to make a reliable decision based on it (Xu et al., 2018). To address this issue, this review focused on the commonality analysis to provide a more inclusive insight considering all the articles including each FRF. However, we did not implement some data from the articles in the pool of review such as sample size, the statistical method, accuracy of the assessment, etc. Hence, having a consistent approach in the future studies on fall risk assessments in stroke survivors and reporting the effect size in a proper format would help the field to get a better and accurate understanding about the impact of various FRFs by providing an inclusive/reliable meta-analysis based on a rich pool of articles in the literature.

**FIGURE 2:**
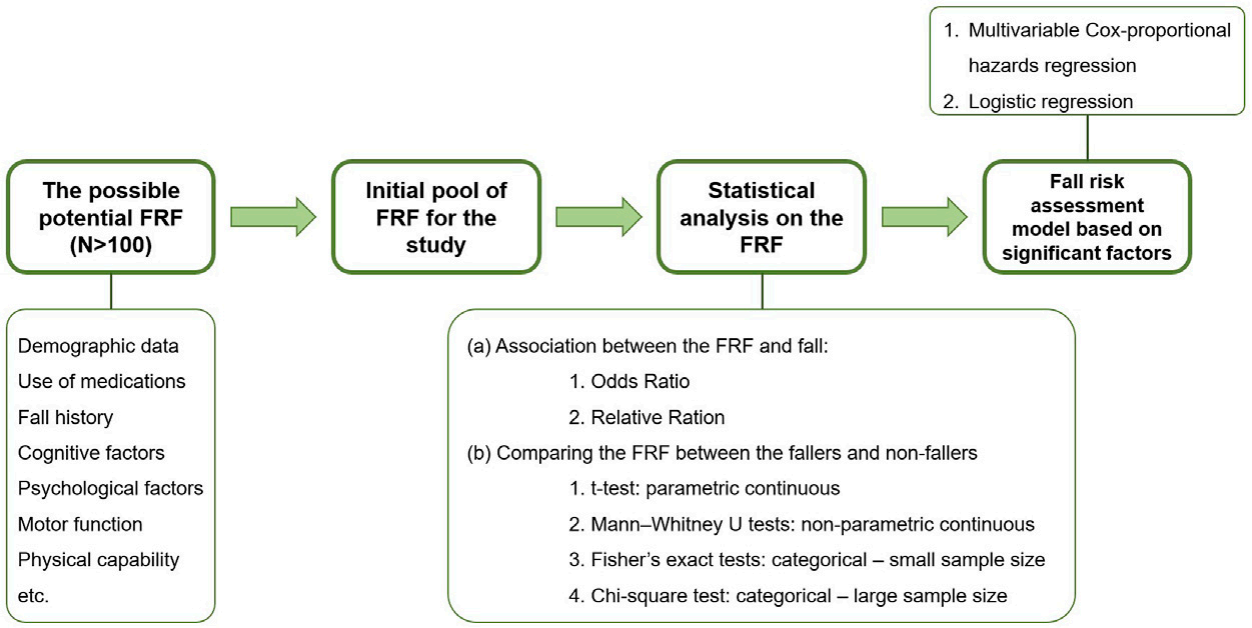
The general path of fall risk assessment studies in stroke survivors with a focus on implemented statistical analysis methods.

There is a significant benefit in providing a review on the statistical methods implemented to analyze the FRFs in the literature. So far, the reviews on the FRFs have been focused on the outcome of the statistical analyses. However, a review of the implemented statistical methods in the studies could help the researchers to compare and select the adequate statistical methods for their analyses. Furthermore, this type of study could investigate the effects of using various statistical approaches/models on the outcome of the fall risk assessment. Such a review study on the methods could be considered as a potential next phase for this systematic review. To this end, a preliminary review was conducted on the articles involved in this review and the statistical methods were summarized in Figure 2. The results of this preliminary review showed that in general, there were six statistical methods implemented in the articles to analyze the effects of each FRF on fall risk. Depending on the type of the analysis and the risk factor, researchers have selected their own set of statistical tools/methods to evaluate the impact of the FRFs on fall risk level. In the next step, in some of the studies, a fall risk assessment model was developed based on the significant FRFs utilizing either multivariable Cox (proportional hazards) regression (e.g., (Foster et al., 2018; Persson et al., 2018)) or logistic regression (e.g., (Lamb et al., 2003; Yoshimoto et al., 2016)). Recently, machine learning approaches such as support vector machine (SVM), multi-layer perceptron (MLP), random forest, decision tree, naïve Bayes, and boosted tree are showing promising results in classifying faller and non-fallers in community dwelling older people (Qiu et al., 2018). Hence, it is highly recommended to explore the capability of these tools to develop fall risk assessment models in stroke community.

According to the results of this review, motion analysis demonstrates a high capability for identifying those at risk for falling. However, obtaining accurate and timely patient information (especially from patients with impaired mobility) is a critical issue which needs to be addressed in future studies. Recent advancements in wearable tools and smartphone technologies, as well as in computational platforms, big data mining and artificial intelligence, have certainly improved options for monitoring and evaluating patients, where several literature reports describe the successful implementation of smartphone motion data to categorize physical status attributes, such as balance or fatigue level (Hou et al., 2018; Karvekar et al., 2020). Similarly, if researchers can validate the capability of a single IMU sensor in fall risk assessment with high accuracy, the developed model could be utilized as a smartphone application. Such an application would reduce the need for clinical visits and provide real-time continuous fall risk assessment data during ADL to clinicians, while engaging patients in self-monitoring and rehabilitation.

## 5 Conclusion

This study presented a systematic review of 27 published papers on fall risk factors and fall risk assessment post stroke, with findings indicating that balance and motion-related measures constitute the most common and significant factors for this at-risk population. Further analysis of these studies demonstrated a clear paradigm shift from using traditional subjective tools to more quantitative objective approaches for assessing balance and motion deficits. Due to the relevance of these two factors in fall risk identification, it is recommended that further studies are needed to investigate an optimal combination of balance/motion assessment tools and protocols for investigating the fall risk for stroke survivors. Considering the accessibility and low cost of high performance IMU sensors, IMU-based analysis, along with other smart sensors, is suggested for capturing motion and balance dynamics. Furthermore, cognitive-motor dual-task studies are highly recommended for future implementation on fall risk assessment of stroke patients for more realistic outcomes. Given the multiple challenges that stroke sufferers face and the critical importance of avoiding additional physical and emotional harm resulting from falls, research targeting the development of advanced fall risk assessment models should be prioritized.

## Data Availability

The original contributions presented in the study are included in the article, further inquiries can be directed to the corresponding author.

## Abbreviations

10MWT, 10 Meter Walk Test; ADL, Activity of Daily Living; BBS, Berg Balance Scale; COP, Center of Pressure; FES, Fall Efficacy Scale; FRF, Fall Risk Factor; FSST, Four Square Step Test; IMU, Inertial Measurement Unit; OR, Odds Ratio; RMS, Root Mean Square; TUG, Timed Up and Go.
